# Chromosome-length genome assembly of the critically endangered Mountain bongo (*Tragelaphus eurycerus isaaci):* a resource for conservation and comparative genomics

**DOI:** 10.1093/g3journal/jkaf109

**Published:** 2025-05-15

**Authors:** Karen M Holm, Klaus-Peter Koepfli, Budhan S Pukazhenthi, Aakrosh Ratan, Karl J Fryxell, Melanie Pham, David Weisz, Olga Dudchenko, Erez Lieberman Aiden, Haw Chuan Lim

**Affiliations:** School of Systems Biology, George Mason University, 10920 George Mason Circle, mail stop 1J1, Manassas, VA 20110, USA; Center for Species Survival, Smithsonian's National Zoo and Conservation Biology Institute, 1500 Remount Road, Front Royal, VA 22630, USA; Department of Biology, George Mason University, Fairfax, VA 22030, USA; Smithsonian-Mason School of Conservation, George Mason University, 1500 Remount Rd, Front Royal, VA 22630, USA; Center for Species Survival, Smithsonian's National Zoo and Conservation Biology Institute, 1500 Remount Road, Front Royal, VA 22630, USA; Department of Genome Sciences, School of Medicine, University of Virginia, PO Box 800717, Charlottesville, VA 22908, USA; School of Systems Biology, George Mason University, 10920 George Mason Circle, mail stop 1J1, Manassas, VA 20110, USA; The Center for Genome Architecture, Department of Molecular and Human Genetics, Baylor College of Medicine, Baylor College, 1 Baylor Plaza, Houston, TX 77030, USA; The Center for Genome Architecture, Department of Molecular and Human Genetics, Baylor College of Medicine, Baylor College, 1 Baylor Plaza, Houston, TX 77030, USA; The Center for Genome Architecture, Department of Molecular and Human Genetics, Baylor College of Medicine, Baylor College, 1 Baylor Plaza, Houston, TX 77030, USA; Center for Theoretical Biological Physics, Rice University, 6100 Main St, Houston, TX 77005, USA; The Center for Genome Architecture, Department of Molecular and Human Genetics, Baylor College of Medicine, Baylor College, 1 Baylor Plaza, Houston, TX 77030, USA; Center for Theoretical Biological Physics, Rice University, 6100 Main St, Houston, TX 77005, USA; Department of Biology, George Mason University, Fairfax, VA 22030, USA; Center for Conservation Genomics, Smithsonian's National Zoo and Conservation Biology Institute, P.O. Box 37012, Washington, DC 20008, USA

**Keywords:** *Tragelaphus eurycerus isaaci*, Bovidae, Hi-C assembly, draft reference genome, conservation, population management

## Abstract

The Mountain bongo (*Tragelaphus eurycerus isaaci*), a critically endangered tragelaphine antelope native to the montane forests of Kenya, faces significant threats from habitat loss and hunting. Although the Mountain bongo is a flagship species in Kenya, the majority are found in small, isolated populations of less than 100 animals total, making it a species of high conservation concern. In this report, we present a chromosome-length draft genome assembly for the Mountain bongo, generated using a combination of linked-read and proximity ligation (Hi-C) sequencing techniques. The assembly resulted in a 2.96 Gb sized genome with a contig N50 of 79.5 kb and a scaffold N50 of 192 Mb. Assembly completeness was 95.1% based on 12,234 Benchmarking Universal Single-Copy Orthologs (BUSCO) and annotation revealed 29,820 protein-coding genes, of which 27,761 were functionally annotated, and a repetitive content of 47.31%. Synteny analysis against the domestic cattle (*Bos taurus*) genome assembly revealed numerous chromosomal rearrangements between the 2 species. Our analysis also revealed insights into the evolutionary and demographic history of the Mountain bongo, offering valuable information for conservation management. We also assembled and annotated the mitochondrial genome which showed <1% differences from the Lowland bongo subspecies, *T. e. eurycerus*. By integrating genomic data with traditional conservation methods, this reference lays the foundation to evaluate and preserve genetic diversity of both in situ and ex situ populations of the Mountain bongo amidst growing environmental pressures.

## Teaser

The Mountain bongo (*Tragelaphus eurycerus isaaci*), a critically endangered antelope native to Kenya, is under threat from habitat loss and hunting. Using a combination of genome sequencing technologies, Holm *et al*. generated the first chromosome-length genome assembly for the species, which they then annotated and evaluated in relation to two previous assemblies of this species. This reference assembly will be used to investigate questions related to genome-wide genetic diversity, genetic structure, inbreeding, and the burden of deleterious variation in both *in-situ* and *ex-situ* populations, thereby providing vital information to inform and guide ongoing conservation efforts.

## Introduction

**Fig. 1.**
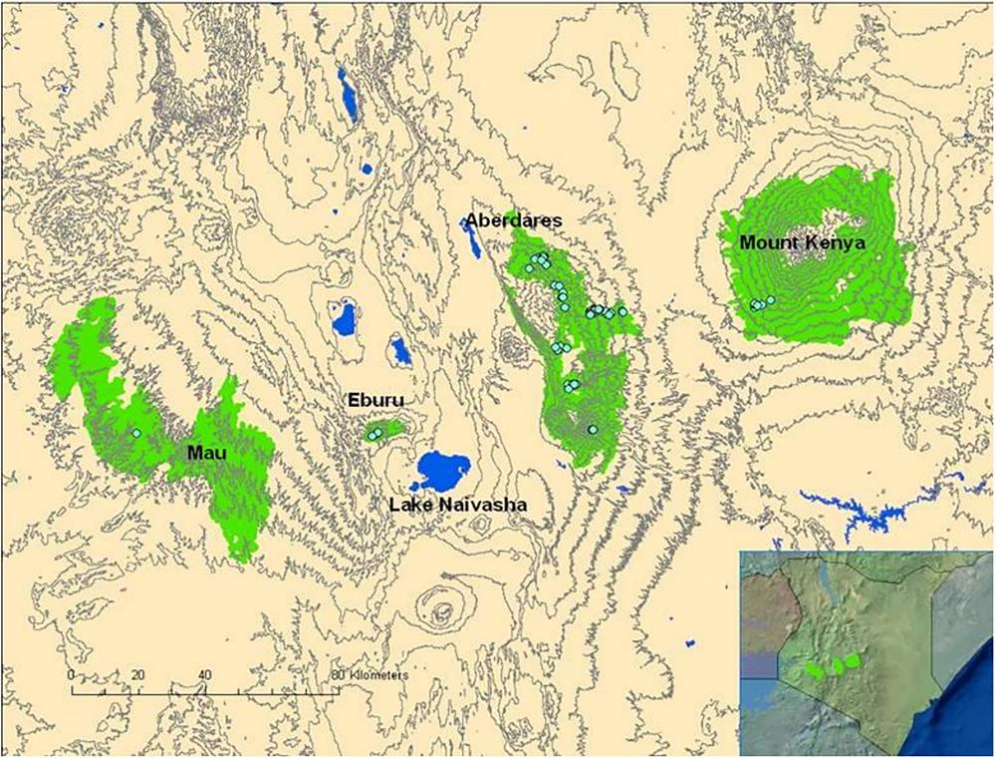
Map of the locations of the four subpopulations of Mountain bongos in Kenya taken from the First Interregional Eastern Bongo Collection Workshop report (original figure sourced from Al Bustan Zoological Centre 2013). Blue dots represent bongo locations in 2013 in the Mau Forest, Eburu Forest, Aberdares Range, and Mount Kenya. Inset shows a satellite map of Kenya with the four forest locations indicated in green.

The Mountain bongo, *Tragelaphus eurycerus isaaci*, (Bovinae: Tragelaphini), a large (>200 kg) spiral-horned antelope native to the montane forests of Kenya, serves as a flagship species for regional conservation efforts. In addition to this eastern subspecies, there is a western or Lowland subspecies (*T. e. eurycerus*) discontinuously distributed in central and western Africa (Gippoliti *et al.* 2018 ). The bongos are 1 of 9 spiral-horned antelope species of sub-Saharan Africa (Rakotoarivelo *et al.* 2024*).* In 2016 the bongo (*T. eurycerus*) was listed as Near Threatened by the International Union for Conservation of Nature Red List of Threatened Species (IUCN SSC Antelope Specialist Group 2017 ), with the number of mature individuals estimated to vary between 15,000–25,000 and a decreasing population trend. However, the *isaaci* subspecies is listed as Critically Endangered with less than 100 remaining in Kenya (IUCN SSC Antelope Specialist Group 2017). This subspecies, divided into 4 populations in Kenya, do not have more than 50 mature individuals (Fig. 1  Al Bustan Zoological Centre, 2013). Their low numbers in the wild are attributed to significant population decline since the 1960s due to habitat loss, hunting, human encroachment and disease (Percival 1928; Elkan and Smith 2013), prompting conservation efforts to include ex situ programs.

Ex situ collections of the Mountain bongo exist in zoos and private breeding facilities worldwide (IUCN SSC, 2014). Estimates of the number of bongos brought into US zoos in the 1960s range from about 20–38 animals and are believed to be imports of the *isaaci* subspecies from Kenya (Bosley 2018; O’Donoghue *et al.* 2017; Gippoliti *et al.* 2018). Today, more than 600–800 bongos reside on ranches mainly in Texas, and approximately 195 are managed in North American zoos under the Association of Zoos and Aquaria Species Survival Plan (AZA SSP), plus several hundreds more internationally (Bosley 2018; Janney 2024). Ex situ populations are managed using different approaches, with some relying on pedigree information (zoos) while others do not (private ranches), which likely influences their genetic diversity and levels of inbreeding. To address the challenges of ex situ management, a reference genome assembly serves as an invaluable resource, enabling the design of genetic markers targeting neutral and adaptive loci for the evaluation of population genomic metrics to assess the genetic health of these populations (Brandies *et al.* 2019; Paez *et al.* 2022).

Our goal was to generate a high-quality chromosome-length reference genome assembly as a foundation for addressing questions related to the genetic status of the Mountain bongo. Previous analysis revealed a karyotype of 2*n* = 33 chromosomes in males and 2*n* = 34 in females (Benirschke *et al.* 1982; Rubes *et al.* 2008). The acrocentric Y chromosome is translocated and fused with the acrocentric autosome 13 (BTA13) causing the imbalance in diploid numbers between the sexes (X^1^X^2^Y system). This provides a cytogenetic framework for our study. Here, we use 10 × Genomics Chromium linked-read sequencing and Hi-C proximity ligation sequencing (Lieberman-Aiden et al. 2009) to produce the first chromosome-length draft reference genome for the Mountain bongo and the genus *Tragelaphus*. We provide genome annotations and analyses of the evolutionary and demographic history of this subspecies. These results will help inform conservation strategies and ensure the persistence of this fascinating species.

## Materials and methods

### Sample collection

**Fig. 2.**
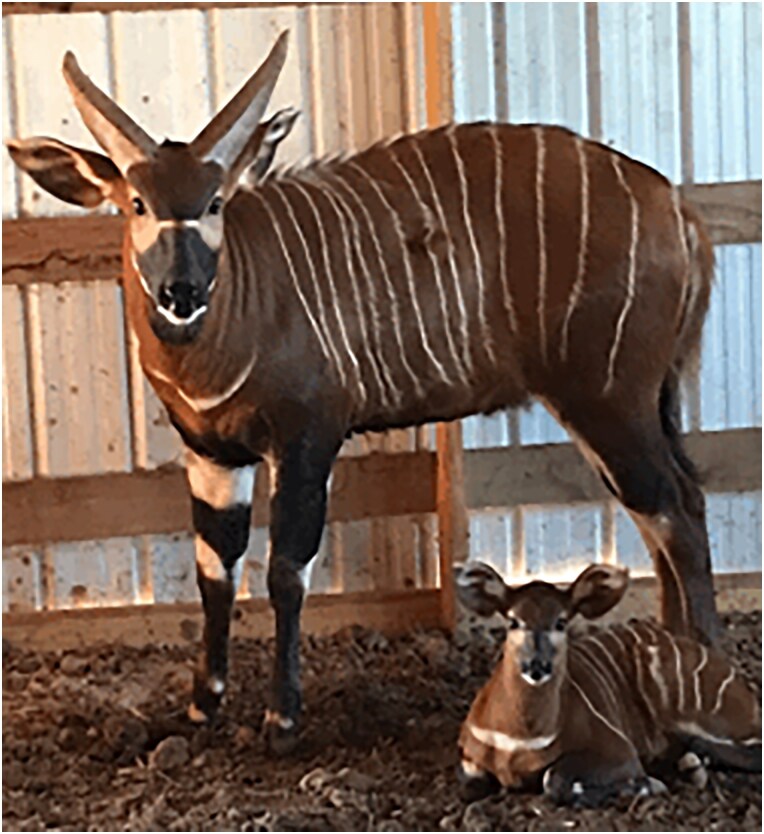
Photograph of Barney (standing), a 12-month old male Mountain bongo at the Wildlife Conservation Center (WCC) in Arrington, Virginia, from which a whole blood sample was collected and used to generate the draft reference genome assembly. Photo credit: David Holm, Director, WCC.

We collected a 4 ml sample of whole blood from a 12-month-old male Mountain bongo (Fig. 2), housed at the Wildlife Conservation Center, a nonprofit breeding center located in Arrington, Virginia (https://wildlifeconservationcenter.org/). During a scheduled transport, the sample was collected opportunistically by venipuncture of the jugular vein and then stored in a 5 ml EDTA Vacutainer blood tube and stored frozen at −20°C until it was shipped on dry ice to Psomagen Inc. (Rockville, Maryland) for DNA processing and 10 × Genomics Chromium linked-read sequencing. The blood sample for the Hi-C scaffolding was provided by the Houston Zoo (USA) from a female Mountain bongo, studbook number 2624. We recognize that using samples from the same individual, or at least the same sex, is common practice. However, differing timelines of sample availability and data generation necessitated the use of samples from different sexes. Furthermore, we focused on assembling the putative autosomal and X chromosome scaffolds, which were unaffected by using samples from different sexes. Nonetheless, using samples from different sexes still allowed us to assemble the autosomes and the X chromosome. Whole blood samples were also collected opportunistically and stored similarly from 4 individuals in North American zoos and ranches (Table 1) to provide us with whole-genome resequencing data for mitochondrial assembly.

**Table 1.** Four resequenced Mountain bongo individuals from North American zoos or ranches and one Central Africa individual used for mitochondrial genome assembly.

North American studbook number (SB), ID or NCBI accession no.	Facility	Type of facility	Sequencing depth
**SB2922**	Memphis Zoo, TN	Zoo	20×
**Bongo 1 (AS)**	Austin Savannah, TX	Ranch	10×
**WCC**	Wildlife Conservation Center, VA	Ranch	10×
**SB2755**	Fossil Rim Wildlife Center, TX	Zoo	10×
**SRR28777087**	Port Lympne Zoo, UK-origin Central Africa	Zoo	8×

Origin is unknown for the UK animal, specifically whether originally from captivity or from the wild (Rakotoarivelo *et al.* 2024).

### DNA extraction, library preparation, and sequencing

Two technologies were used to produce data for the draft reference genome. First, the scaffolded assembly was generated using data from 10 × Genomics Chromium linked-read sequencing. High molecular weight DNA (50–100 kb) was isolated from an aliquot of whole blood using the MG Blood Genomic DNA Extraction SV kit (MGmed; Seoul, Korea) in combination with Qiagen Genomic-tips, according to the manufacturer's protocol. Extracted genomic DNA fragment lengths were evaluated and quality checked using an Agilent 4200 Tapestation (Agilent Technologies, Santa Clara, CA, USA). The genomic library was built using DNA fragments captured in Gel Bead-In-Emulsions and nick-translated using bead-specific unique molecular identifiers in a 10 × Genomics Chromium Controller instrument fitted with a microfluidic Genome Chip according to manufacturer protocols (Chromium, 2018). The prepared library was then paired-end sequenced (150 bp) on 2 lanes of an Illumina NovaSeq6000 instrument with S4 flow cells. Peripheral blood mononuclear cells collected from the female bongo (studbook number 2624) were used to prepare 2 in situ Hi-C libraries. Library preparation followed the protocols in Rao *et al.* (2014). The libraries were pair-end sequenced (2 × 150 bp) on an Illumina HiSeq X Ten instrument at the Baylor College of Medicine (Houston, Texas).

To generate whole-genome sequencing data for the mitochondrial genome assemblies, the 4 whole blood samples were sent to Psomagen Inc. (Rockville, Maryland) for DNA extraction, library preparation using the TruSeq DNA PCR-free library kit (Illumina, San Diego, CA, USA) and paired-end sequencing (2 × 150 bp) at 10–20 × depth on an Illumina NovaSeq6000 instrument S4 flow cells.

### Genome assembly and completeness

#### Assembly

The raw reads generated from the 10 Genomics Chromium library were quality checked using FastQC v 0.90 (Andrews 2010). Approximately 1.1 billion reads were then assembled into partially phased pseudo-haplotypes using the Supernova Assembler v 2.1.1 (Weisenfeld *et al.* 2017). This number of reads and genome depth of coverage (∼56× ) were chosen in keeping with recommendation provided by Weisenfeld *et al.* (2017). The Hi-C data was aligned to the Supernova assembly using Juicer v1.6 (Durand *et al.* 2016). The Hi-C-guided scaffolding was performed using the 3D-DNA pipeline (Dudchenko *et al.* 2017). The resulting assembly was reviewed and curated using Juicebox Assembly Tools v2020 (Dudchenko *et al.* 2018). Hi-C contact maps before and after the Hi-C-guided scaffolding were visualized using Juicebox (Durand *et al.* 2016). The assembly comprising Supernova scaffolds and scaffolded with Hi-C data is hereafter referred to as the “assembly”. Assembly statistics were calculated in QUAST v 5.2.0 (Mikheenko *et al.* 2018) and a custom Python script (*assembly_stats.py*) was used to determine the contig N50. We did not assemble the Y chromosome due to its high repetitive sequence content and difficulty in assembly using short-read sequencing technology. In fact, very few mammals and no member of the *Bovidae* family have had the Y chromosome assembled until very recently (Olagunju *et al.* 2024).

#### Completeness

To assess completeness of the assembly, we ran Benchmarking Universal Single-Copy Orthologs (BUSCO) v5.7.0 (Manni *et al.* 2021). This tool uses hidden Markov models to detect homologs with hmmsearch 3.1 and gene discovery using the default MetaEuk: 4.a0f584d (Manni *et al.* 2021; Zdobnov *et al.* 2021). We used the laurasiatherian_odb10.2019-11-20 lineage database available at https://www.orthodb.org (Kriventseva *et al.* 2019), which contains 12,234 single-copy orthologs from 52 species.

### Genome annotation and synteny

#### Repetitive elements

To structurally annotate the Mountain bongo genome, we identified the repetitive element content within the assembly. First, we ran RepeatMasker v4.1.6. using the *Bovidae* database within Dfam 3.8 (Storer *et al.* 2021). We also ran RepeatMasker using the *Tragelaphus* library, available for download from the Dfam repository (www.dfam.org), to compare the results. Soft masking of these repetitive elements, including low-complexity sequences and interspersed repeats, or transposons, was implemented by replacing these regions with lowercase letters rather than Ns or Xs (Tarailo-Graovac and Chen 2009; Smit, Hubley and Green 2015; Storer *et al.* 2021; RepeatMasker at http://repeatmasker.org).

#### Annotation

Following soft masking of the assembly, we executed gene prediction by means of the homology-based Gene Model Mapper pipeline (GeMoMa) v1.9 (Keilwagen *et al.* 2018) using the following flags: AnnotationFinalizer set to “no” so as not to rename genes or transcripts, GeMoMa_score set to ReAlign to realign the search results, and o = true to return predictions for each species. All other parameters were set to the defaults. We used reference genomes of 6 species for annotation: *Homo sapiens* (genome version: GRCh38.p14; NCBI accession number GCA_000001405.40; Fain *et al.* 2019); *Bos taurus* (ARS-UCD2.0; GCF_002263795.3; Rosen *et al.* 2020); *Bison bison bison* (Bison_UMD1.0; GCF_000754665.1; Dobson *et al.* 2021); *Orcinus orca* (mOrcOrc1.1; GCF_937001465.1; Foote and Bunskoek 2022); *Capra hircus* (ARS1.2; GCF_001704415.2; Bickhart *et al.* 2017); and *Ovis aries* (ARS-UI_Ramb_v3.0; GCF_016772045.2; Davenport *et al.* 2022). We extracted gene models from these species using tblastn v1.6.4 (Camacho *et al.* 2009) followed by the GeMoMa Annotation Filter to filter redundant and low-quality predictions which then selects and extracts the relevant predictions. We then assessed the annotation with GenomeQC using the mammalia_odb9 database (Manchanda *et al.* 2020). Finally, we functionally annotated the protein-coding genes (PCGs) using the online version of eggNOG-mapper v2.1.12 (Huerta-Cepas *et al.* 2019; Cantalapiedra *et al.* 2021), which used the Diamond v2.1 search database iteratively (Buchfink *et al.* 2021), with all other parameters set to default.

#### Synteny assessment

To assess large-scale structural variation and genomic synteny against a related species, we aligned the 17 chromosome-length scaffolds (all scaffolds > 20 Mb in size) of the bongo assembly to the 29 autosomes and the X chromosome of the domestic cattle (*Bos taurus*) genome assembly (BosTau9; GCA_002263795.4; Zimin *et al.* 2009). This cattle assembly is one of the most high-quality, contiguous, and well-annotated genome available from the family *Bovidae* and is thought to represent the ancestral bovid karyotype (Rosen *et al.* 2020). It has been used multiple times for synteny comparisons in other antelope species, including sable, scimitar-horned oryx, and roan (Koepfli *et al.* 2019; Humble *et al.* 2020; Gonçalves *et al.* 2021, respectively). We performed the analysis using Satsuma2 (Grabherr *et al.* 2010) which uses an alignment process that finds sequence matches through cross-correlation of homologous DNA regions that have diverged over time. We then used the script *BlockDisplaySatsuma* within Satsuma2 to create a synteny block file to plot these differences in R v4.4.1 (R Core Team 2024) using the package gggenomes extension (Hackl *et al.* 2024) in the ggplot2 package (Wickham 2016).

### Mitochondrial genome assembly and phylogenetic analysis

After trimming adapters using TrimGalore! v0.6.4 (Krueger 2021) from the 4 whole-genome resequenced samples, the mitochondrial genome was assembled for each using MitoZ v3.6 with SPAdes v3.15.5 and Megahit v1.2.9 as the assemblers, as well as Tiara v1.0.3 and HMMR v3.1b2–3 for scaffolding with default *k*-mer values (Wheeler and Eddy 2013; Xie *et al.* 2014; Li *et al.* 2015; Nurk *et al.* 2017; Chen *et al.* 2018; Meng *et al.* 2019; Karlicki *et al.* 2022). The assembled mitogenomes were then annotated within MitoZ v3.6 using tblastn within Blast + v2.15.0 (Gertz *et al.* 2006), Genewise within wise2 v2.4.1(Birney *et al.* 2004), and Infernal v1.1.1 (Nawrocki and Eddy 2013). We used the same methods to assemble and annotate the mitochondrial genome of a Central African bongo sample collected from Port Lympne zoo (United Kingdom) after downloading the whole-genome read data from the NCBI accession number SAMN41052124, SRR28777087; Rakotoarivelo *et al.* 2024.

To determine the phylogenetic placement of the newly assembled Mountain bongo and Central African bongo mitogenomes within the tribe *Tragelaphini*, we generated a multisequence alignment that included 9 additional *Tragelaphus* species. This dataset also included the Lowland bongo (*T. e. eurycerus)*, 3 isolates from the bushbuck (*T. scriptus*), and the African buffalo (*Syncerus caffer)* as the outgroup (Supplementary Table 1). All sequences were downloaded from GenBank (Hassanin *et al.* 2012; Supplementary Table 1) and aligned using the MAFFT v7.490 alignment plugin within Geneious Prime 2021.2 (https://www.geneious.com) with the following settings: gap open penalty = 1.53 with offset value 0.123, scoring matrix 200PAM/k = 2 and automatically determining sequences’ direction (Katoh and Standley 2013). After excluding the control region due to poor alignment with the tandem repeat regions, we then built a maximum likelihood tree using the web version of IQ-TREE (W-IQ-TREE, Trifinopoulos *et al.* 2016; http://iqtree.cibiv.univie.ac.at) by means of the auto function ModelFinder to determine the best-fitting DNA substitution model for our data (Kalyaanamoorthy *et al.* 2017). We calculated node support with the ultrafast bootstrap analysis set at 1,000 iterations (Hoang *et al.* 2018) and performed single branch tests using SH-like approximate likelihood ratio tests (SH-aLRT) for internal branch support (Guindon *et al.* 2010).

### Demographic history

To reconstruct the demographic history of the Mountain bongo, we carried out pairwise sequentially Markovian coalescent (PSMC) analysis using PSMC v.0.6.5 (Li and Durbin 2011). First, we mapped the 10 × Genomics Chromium linked-reads back to the assembly using BWA-MEM v.0.7.17 (Li and Durbin 2009). Next, we removed the BAM file corresponding to the X chromosome (Hi-C_Scaffold_17) and all BAM files associated with small scaffolds < 20 Mb in size. The sex chromosomes were excluded because they are haploid and PSMC requires diploid sequences (Li and Durbin 2011). Additionally, their distinct mutation and recombination rates along with differences in Mendelian inheritance patterns compared to autosomes, could bias estimates of effective population size (N_e_) and demographic history inferences. This allowed us to create a consensus sequence using BCFtools v1.19 mpileup and vcfutils.pl with the -C50 option to apply a coefficient to adjust the base alignment quality (Danecek *et al.* 2021). We then ran PSMC with the following parameters -p “4 + 25*2 + 4 + 6” and with 100 bootstraps replicates. To plot changes in effective population size through time, we used a generation time of 5 years and a mutation rate of 1.1e−08 per site per generation (Chen *et al.* 2019).

## Results and discussion

### Genome assembly and completeness

#### Assembly

**Fig. 3.**
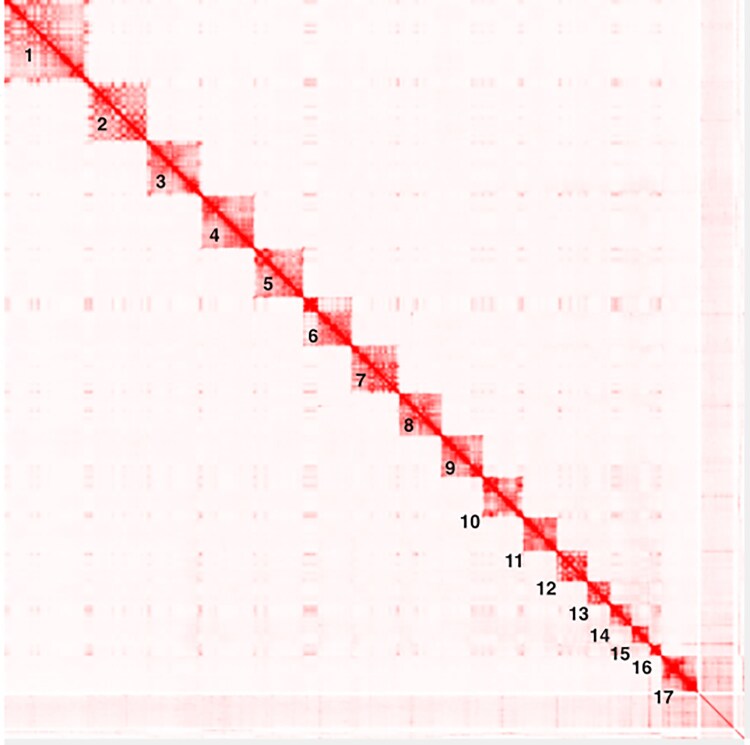
Contact map showing the Hi-C data aligned to the Mountain bongo chromosome-length genome assembly. The contact map shows, on the scale from white to red, the frequency of Hi-C contacts between loci across the genome. The 17 squares along the diagonal mark are the 17 chromosome-length scaffolds (Hi-C_scaffold_X), labeled 1–17. Interactive version of this map is available at https://www.dnazoo.org/assemblies/tragelaphus_eurycerus_isaaci.

Illumina sequencing of the 10 × Genomics Chromium libraries produced ∼497 Gb or 3.3 × 10^9^ paired-end reads, with a raw genomic coverage of 171 × and an effective coverage of 57.6 × coverage as estimated by the Supernova v2.1.1 assembler. We outputted the Supernova assembly in the partially phased pseudo-haplotype style. We chose this style because our downstream analyses do not require phased or haplotype-resolved data. This style provides a simplified, representative reference assembly suitable for read mapping and population-level variant analysis, while avoiding the added complexity of retaining both haplotypes. The Supernova assembly had a total length of 2.8 Gb while the scaffolded assembly was 2.96 Gb in length based on the QUAST output (Table 2). The linked-read technology facilitates linking of reads from the same long DNA molecule and the key measure of the Supernova assembly quality is scaffold N50 as well as other scaffold-based metrics. This assembly had a scaffold N50 of 13.7 Mb with 36,966 scaffolds (4,811 scaffolds > 10 kb). The Hi-C scaffolded assembly was significantly more contiguous, with a scaffold N50 of 192 Mb and a maximum scaffold size of 0.34 Gb. The GC content was 41.97%. The final assembly organized scaffolds into 17 pseudo-chromosomes comprising 16 autosomes and the X chromosome (Fig. 3), increasing the scaffold N50 ∼14-fold relative to the Supernova assembly. This genome assembly is 9 times more contiguous in terms of scaffold N50 than the assembly generated using Oxford Nanopore Technology (ONT) long reads (mTraEur1.1; NCBI accession number GCA_935064755.1) (Table 2).

**Table 2.** Assembly metrics and BUSCO scores (based on laurasiatheria odb10 lineage gene set containing 52 species and 12,234 genes) of the Mountain bongo draft genome reported in this study in comparison to two other genome assemblies for the bongo.

*Assembly Statistic*	*Current Assembly*	*GCA_935064755.1 mTraEur1.1*	*GCA_006410755.1 BNG*
*# contigs (≥0)*	35382	1863	1539703
*# scaffolds*	16784	1562	206316
*Largest scaffold*	337452246	79316757	205988
*Total length*	2.94 Gb	2.84 Gb	2.42 Gb
*GC %*	41.97	41.97	41.43
***Contig N50***	**0.08 Mb**	**21.2 Mb**	**2 kb**
***Scaffold N50***	**192 Mb**	**21.2 Mb**	**0.016 Mb**
*N90*	86,146,650	3,066,938	5423
*L50*	7	38	44988
*L90*	15	152	147,210

*BUSCO scores*	*% (total number)*	*% (total number)*	*% (total number)*
*COMPLETE*	95.10% (11628)	98.5% (12045)	48.4% (5926)
*Complete and Single-copy*	91.40% (11181)	95.8% (11717)	47.8% (5851)
*Complete and duplicated*	3.70% (447)	2.7% (328)	0.6% (75)
*Fragmented*	1.30% (157)	0.8% (92)	23.2% (2843)
*Missing*	3.60% (449)	0.7% (97)	28.4% (3465)

Assembly metrics were estimated using QUAST v5.2.0. Totals in numbers (#) or total length in number of millions of base pairs (Mb). The contig N50 values were produced from the custom python script, assembly_stats.py.

#### Completeness

Benchmarking Universal Single-Copy Ortholog (BUSCO) scores indicated high completeness of the assembly, (95.1% or 11,181 out of 12,234 complete and single-copy BUSCOs) (Table 2). There was a marginal improvement of the completeness in the ONT assembly (mTraEur1.1) compared to this assembly (98.5%) and the contig assembly (BNG) was significantly less complete (48.4%) (Table 2). Similarly, the Hi-C assembly of the scimitar-horned oryx (*Oryx dammah*) revealed a 93.3% completeness in single-copy and duplicated BUSCOs (Humble *et al.* 2020). The completeness of the Mountain bongo genome compares favorably with other ungulate genome sequences (Supplementary Table 2).

### Genome annotation and synteny

#### Repetitive elements

Using the curated *Bovidae* library within RepeatMasker on the assembly revealed 48.53% bases masked as repetitive regions, while using the *Tragelaphus-*specific library produced 47.31% total masked bases (Tables 3 and 4, respectively). The most abundant repetitive elements were long-interspersed elements (LINEs) from the Bov-B-LINE groups, respectively accounting for 14.72% out of 29.37% and 16.51% out of 29.97% total LINEs in each database. This is similar to the number of Bov-B LINEs in the okapi (*Okapi johnstonoi*) *TBG_Okapi_asm_v1* assembly (15.27%) (Winter *et al.* 2022). Horizontal transfer of Bov-B LINEs from Squamata, specifically vipers (Viperadae) to Ruminantia, occurred in the distant past and are now seen in many vertebrate lineages (Kordiš and Gubenšek 1999; Ivancevic *et al.* 2018). The amount and type of repetitive elements in the bongo are similar to other ungulate species (Supplementary Table 2).

**Table 3.** RepeatMasker from this assembly of the Mountain bongo using the curated Bovidae repeat database.

*Element*	*Number of elements*	*Length occupied*	*Percentage of sequence*
*Retroelements*	4291777	1346034687 bp	45.36%
* SINEs:*	2232439	354429003 bp	11.94%
* LINEs:*	1647560	871397545 bp	29.37%
* RTE/Bov-B*	671228	436693209 bp	14.72%
* LTR elements:*	411778	120208139 bp	4.05%
*DNA transposons*	308889	60038093 bp	2.02%
*Unclassified:*	16019	1576063 bp	0.05%
*Total interspersed repeats*	—	1407701472 bp	47.44%
*Small RNA:*	297156	41876555 bp	1.41%
*Satellites:*	1	326 bp	0.00%
*Simple repeats:*	524837	21404868 bp	0.72%
*Low complexity:*	82335	4004089 bp	0.13%
*Total masked*	*—*	*1440218448 bp*	*48.53%*

**Table 4.** RepeatMasker results from this assembly of the Mountain bongo using the Tragelaphus repeat library and partially hand-curated with the ancestral elements as well.

*Element*	*Number of elements*	*Length occupied (bp)*	*Percentage of sequence* (%)
*Retroelements*	3541715	1300030002	43.81
* SINEs:*	1640428	297166967	10.01
* LINEs:*	1580483	889590460	29.98
* RTE/Bov-B*	724270	489887153	16.51
* LTR elements:*	320804	113272575	3.82
*DNA transposons*	283294	58496467	1.97
*Unclassified:*	4359	1145224	0.04
*Total interspersed repeats*	—	1359683715	45.82
*Small RNA:*	266696	46229246	1.56
*Satellites:*	7707	18453152	0.62
*Simple repeats:*	516182	21157359	0.71
*Low complexity:*	80273	3895637	0.13
*Total masked*	*—*	*1403894046*	*47.31*

#### Annotation

Gene prediction and annotation of the reference assembly resulted in 29,820 PCGs with 27,761 of these functionally annotated. This number of PCGs is similar to that found in the domestic cattle 30,589 (*Bos taurus*; Zimin *et al.* 2009) and to other antelope species: 30,622 in the roan antelope (*Hippotragus equinus;* Gonçalves *et al.* 2021), and 28,720 in the scimitar-horned oryx (*Oryx dammah)*, of which 21,161 were functionally annotated (GCF_014754425.2; Humble *et al.* 2020). However, the number of PCGs annotated in the Mountain bongo are appreciably higher than in other antelope species: 21,276 in sable (*Hippotragus niger*; Koepfli *et al.* 2019) and 23,125 in gemsbok (*Oryx gazella*; Farré *et al.* 2019). The number of annotated PCGs can vary not only by species but can vary depending on sequence coverage, assembly method, and the quality of assembly, which can potentially introduce bias (de Sá *et al.* 2018). Additionally, different annotation approaches have idiosyncratic strengths and limitations. For example, while ab initio methods do not require external data, they can produce false positive and less accurate gene structures (e.g. AUGUSTUS; Stanke and Morgenstern 2005). In contrast, methods that use extrinsic evidence usually produce very accurate gene models but are optimal when evidence, such as RNA-seq data, is collected from multiple tissues (e.g. Maker2; Holt and Yandell 2011). Lastly, homology-based prediction methods produce high-accuracy gene models when good quality annotations from reference genome assemblies of related species are available, as seen with GeMoMa in the present study (Keilwagen *et al.* 2018).

#### Synteny

**Fig. 4.**
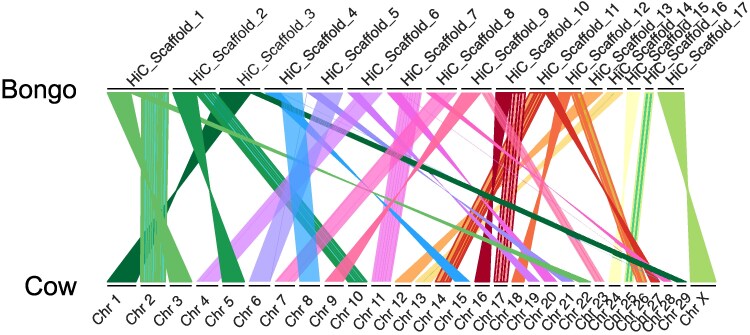
Chromosomal synteny between the Mountain bongo (*Tragelaphus eurycerus isaaci*) and domestic cattle (*Bos taurus*) showing scaffolds larger than 20 Mb (chromosome-length, Hi-C_Scaffold 1–17) in the bongo relative to the domestic cattle chromosomes (29 autosomes and the X chromosome). Scaffolds were ordered to obtain the best pairwise alignment blocks between the two genomes which are visualized using colored lines. Hi-C_Scaffold_17 in the Mountain bongo was inferred to be the X chromosome based on direct synteny with Chr X in the cattle.

Visualization of genomic synteny between the Mountain bongo (2*n* = 33 in males, 2*n* = 34 in females) and domestic cattle (2*n* = 60) revealed the bongo has undergone a significant number of the chromosomal fusion events compared to domestic cattle. These fusion events are notably more pronounced in Mountain bongo than in other species within Bovidae, such as the gemsbok (Farré *et al.* 2019), roan antelope (Gonçalves *et al.* 2021) or scimitar-horned oryx (Humble *et al.* 2020), which show a closer chromosomal alignment and higher synteny with domestic cattle, due to more similar chromosome numbers (2*n* = 60 in roan and sable antelope, 2*n* = 56–58 in the scimitar-horned oryx; Graphodatsky *et al.* 2020). Twelve of the 17 chromosome-length scaffolds in the Mountain bongo assembly each correspond to syntenic regions spanning 2–3 chromosomes in the cattle genome. For example, bongo Hi-C_scaffold _1 corresponds to 3 cattle chromosomes, Chr 2, Chr 3, and Chr 22 (Fig. 4). Similarly, Hi-C_scaffold_17 in the bongo corresponds to the X chromosome in cattle. The number of chromosomes varies in the *Tragelaphini* tribe from 2*n* = 30 to 2*n* = 57/58 as a result of chromosome evolution and is thought to play a role in reproductive isolation (Rubes *et al.* 2008; Robinson and Ropiquet 2011; Rakotoarivelo *et al.* 2024). *Bovidae* have a high number of Robertsonian fusions and chromosomal rearrangements which may account for this variation (Gallagher and Womack 1992). Most *Tragelaphini* species also possess an acrocentric Y-autosomal translocation involving BTA 13 resulting in sex-specific imbalance in diploid chromosomes. Female bongos possess 1 of 2 distinct morphotypes of the X chromosome characteristic of the X^1^X^2^Y sex chromosome system (Benirschke *et al.* 1982; Rubes *et al.* 2008).

### Mitochondrial genome assembly and phylogenetic analysis

**Fig. 5.**
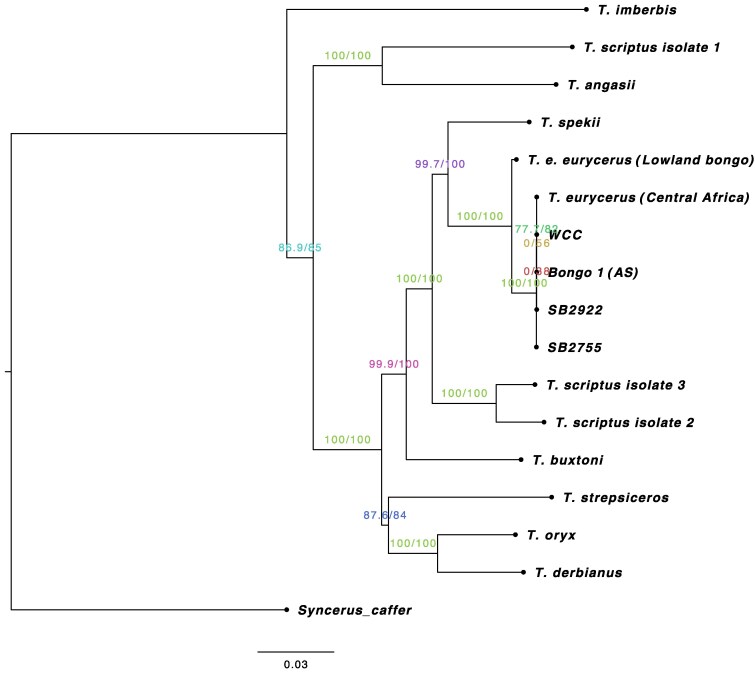
Maximum likelihood phylogenetic tree generated from a 15,843 bp alignment (excluding the control regions) of the mitochondrial genomes of 10 *Tragelaphus* species including 4 Mountain bongos, one Central African bongo and one Lowland bongo. The African buffalo (*Syncerus caffer)* was used as the outgroup to root the tree. Numbers on internal branches are SH-aLRT support (%)/ultrafast bootstrap support (%) value and indicate node support. Branch lengths are proportional to number of substitutions per site (see scale bar).

The length of the Mountain bongo mitochondrial genome is 16,348 bp with a GC content of 39.4%, similar to that of the Lowland subspecies (16,349 bp) as well as most mammalian species (Boore 1999). We annotated the mitogenomes of the 4 whole-genome resequenced samples using MitoZ, identifying a total of 37 genes, including 13 PCGs, 2 rRNAs, and 22 tRNAs. Only 0–3 single nucleotide differences (0.024%) were observed between the Mountain bongos and the Central African bongo. However, there were 197–202 nucleotide differences identified between the Mountain and the Lowland subspecies, representing less than 1% divergence. Additionally, there were 95 nucleotide differences between the Central African bongo and the Lowland subspecies. Among 81 codon changes, 18 (∼22%) were non-synonymous substitutions, while 63 (∼78%) were synonymous. Notably, the 18 substitutions occurred in each the *CYTB* (2/9), *ATP6* (3/8)*, ATP8* (2/2), *COX3* (2/5)*, ND2* (1/6) *ND3* (1/3), *ND4* (1/9), *ND5* (5/12), and *ND6* (1/3) genes. All substitutions occurring in the *COX1* (12), *COX2* (2), *ND1* (10) genes were synonymous. The majority of single nucleotide differences were transitions. There are fewer differences between these bongo subspecies than there are among the 3 different bushbuck isolates (*Tragelaphus scriptus*) reported in Hassanin *et al.*, (2012) that are now considered subspecies (Rakotoarivelo *et al.* 2024). Using a final alignment of 15,843 bp, a maximum likelihood phylogenetic reconstruction was performed with the inferred TIM2 + F + I + G4 model as the best-fitting substitution model. The tree shows that the Lowland bongo and Mountain + Central African bongos constitute distinct haplotypes and that bongos are sister to the amphibious sitatunga (*Tragelaphus spekii*), which ranges throughout Central Africa (Fig. 5). Overall, the mitochondrial genome topology is consistent with previous findings (Hassanin *et al.* 2012; Rakotoarivelo *et al.* 2024). To the best of our knowledge, the Mountain bongo mitogenome assemblies are the first to be reported for this subspecies.

### Demographic history

**Fig. 6.**
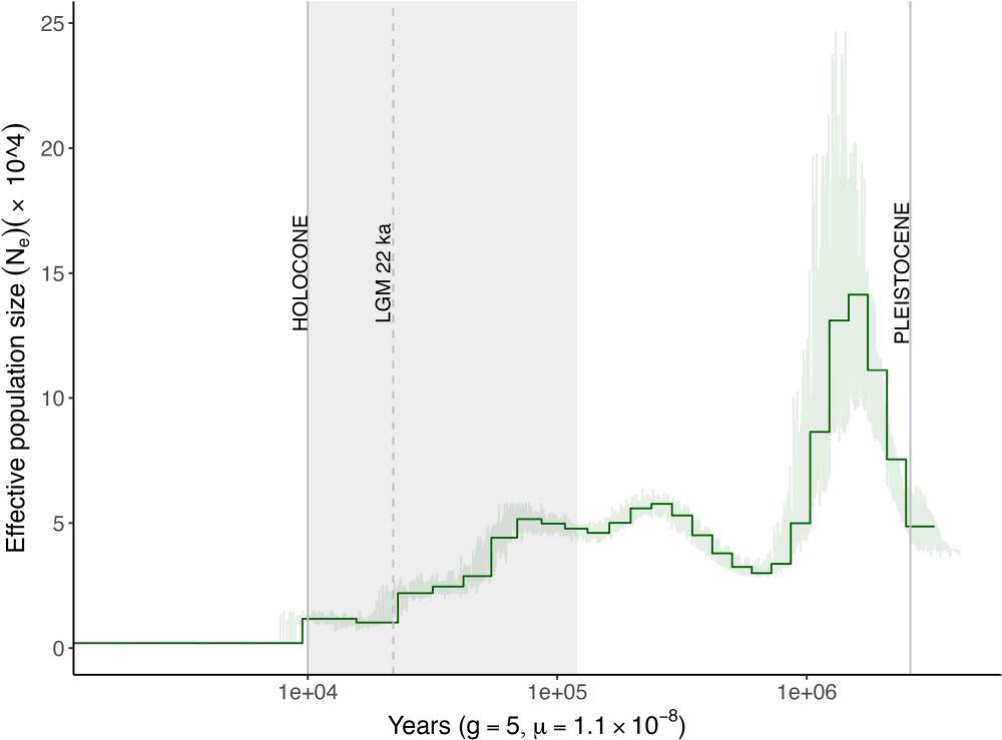
Demographic history determined by PSMC using a generation time of 5 years and a mutation rate of 1.1 ×10^−8^ per site per generation. Thick green line represents the reference genome with 100 bootstraps in light green. The gray shaded area corresponds to the Last Glacial Period or Ice Age, dashed line Last Glacial Maximum at 22,000 years (Bowen 2009).

The estimation of effective population size (N_e_) over time was characterized using PSMC (Fig. 6). The trajectory was similar to that reported in Chen *et al.* (2019) in that there was an expansion from about 1 million years ago to 100,000 years ago followed by a population decline. However, our estimate of N_e_ was 2–3 × higher than the estimate in Chen *et al.* (2019). This discrepancy could be due to significant differences in the genome assembly (contig N50 = 1.98 kb and no scaffolds) of the bongo genome that was used in Chen *et al.* (2019) (Mather *et al.* 2020). The bongo experienced a continuous population decline until approximately 20,000 years ago, likely due to anthropogenic factors, as montane forests diminished and human populations grew (Bae *et al.* 2017). Climatic changes during the late Pleistocene and Holocene (10–15 kya) led to forest expansion in the Mau Forest Complex, creating suitable habitats for the bongo (Githumbi *et al.* 2021). Despite this, bongo populations continued to decline, likely due to human expansion and climate change during the Last Glacial Maximum. Combined effects of these fluctuations in climate and human encroachment drove many ungulate extinctions (Barnosky *et al.* 2004; Hernández Fernández and Vrba 2006; Hoag and Svenning 2017). The bongo's demographic trajectory is similar to other forest-dwelling antelope species, for example the sitatunga (*T. Spekii*) and the bushbuck (*T. scriptus*) (Chen *et al.* 2019). However, Chen *et al.* (2019) observed in their multispecies PSMC analysis that patterns did not clearly align with either habitat or feeding type.

## Conclusions and conservation implications

The current de novo reference genome for the Mountain bongo (*Tragelaphus eurycerus isaaci*) is the first annotated chromosome-length assembly produced for the genus *Tragelaphus*. Our assembly is highly contiguous at the scaffold level compared to other contig-length assemblies available for this species, although it has lower contiguity at the contig level when compared to an assembly generated using Oxford Nanopore Technologies (ONT) long reads (GCA_935064755.1). Our assembly has been resolved structurally using the Hi-C scaffolding at the chromosome level, which is an improvement compared to other bongo assemblies published. We generated functional annotations of PCGs from the reference assembly as well as the mitochondrial genome assemblies. Synteny comparison with the domestic cattle (*Bos taurus*) genome showed a high number of chromosomal rearrangements in the bongo genome. As the ancestral karyotype of the Bovidae has been computationally reconstructed as 2n = 60 (Damas *et al.* 2022), this suggests that the bongo karyotype (2*n* = 33 or 34) has changed primarily through chromosomal fusion events. For the mitochondrial genome assemblies, we utilized whole-genome resequenced individuals, as linked-read DNA sequencing, which incorporates size selection of large (>50 kb) DNA molecules, is less effective for assembling small, high-copy-number genomes like mitochondria due to its dependence on long-range contiguity and limited barcoding coverage in these regions. Although an ideal comparison would include multiple samples of the Lowland bongo, the acquisition of such samples remains challenging due to political and logistical barriers in western Africa; however, museum samples could be potentially utilized. At a larger scale, the Mountain bongo genome broadens our understanding of mammalian and ungulate genomes, especially within the genus *Tragelaphus*. A series of highly contiguous genomes will enable future studies on gene or gene family evolution, as well as the detection of gene loss/gain and structural variants, providing a critical resource for comparative genomics.

Most importantly, our genome assembly provides a foundation for future management of both in situ and ex situ populations of the Mountain bongo, with applications including, but not limited to, monitoring genetic diversity, assessing inbreeding levels, identifying adaptive traits, and estimation of relatedness. Small populations, such as those in human care (zoos, ranches, or breeding centers) or fragmented wild populations, are at significant risk of erosion of genetic diversity, as well as increased inbreeding and possible inbreeding depression (Frankham *et al.* 2010). These genetic consequences can make bongos more susceptible to environmental stressors and diseases, exacerbating their vulnerability. Understanding these genetic threats through in-depth analysis is critical for identifying where genetic diversity stands in ex situ populations, which is vital for maintaining an insurance population or planning potential reintroductions (IUCN/SCC, 2014; Hoban *et al.* 2021).

Moreover, access to a reference genome and its annotations supports the development of genetic tools for managing genetic health, such as SNP genotyping panels for target-capture sequencing, to aid in the management of both in situ and ex situ populations worldwide, similar to efforts made with the sable antelope, *Hippotragus niger* (Koepfli *et al.* 2019; Gooley *et al*. 2020) and European bison, *Bison bonasus* (Wehrenberg *et al.* 2024). In addition, predicting deleterious mutations in breeding programs could help minimize the impacts of inbreeding depression (Robinson *et al.* 2019; Hohenlohe *et al.* 2021), while monitoring the accumulation of runs of homozygosity and screening for variants linked to disease resistance (Fitak *et al.* 2019) could help identify genetically healthier individuals for reintroduction or breeding. By facilitating research into the species’ evolutionary history and informing conservation strategies, this genome represents a crucial step toward ensuring the persistence of this flagship species in the face of numerous challenges within its native habitat.

## Supplementary Material

jkaf109_Supplementary_Data

## Data Availability

The 10 × Genomics Chromium linked-read raw sequence data are available on NCBI under Bioproject PRJNA1081906, NCBI SRA accession number SRR32264120. The individual used for Hi-C scaffolding is available on NCBI: Biosample SAMN21582269; Hi-C data SRR16086839 and SRR16086840. The Supernova + HiC assembly and annotations are available on NCBI under BioProject PRJNA1081906 and accession number JBBXMO000000000. The 4 samples of whole-genome resequenced raw reads for the mitochondrial genomes are available under NCBI BioProject PRJNA1220685. The mitochondrial genome assemblies and annotations are available on NCBI under the same BioProject and the following GenBank accession numbers: PV619107 (SB2922), PV619107 [bongo 1 (AS)], PV619108 (SB2755), PV619109 (WCC). The annotations for the assembly are available at GSA FigShare: https://doi.org/10.25387/g3.28373795. Computational scripts for the Supernova assembly are included in supplemental methods in Supplementary Material. The methods and scripts for the Hi-C scaffolding are available on the following website: https://www.dnazoo.org/methods, cited in the following references (Dudchenko *et al.* 2018, 2017) with corresponding GitHub repositories. Supplemental material available at G3 online.
