# Supplementary material for: Chromosome-length genome assembly of the critically endangered Mountain bongo (Tragelaphus eurycerus isaaci): a resource for conservation and comparative genomics
Source: G3 (Bethesda). 2025 May 15;15(7):jkaf109. doi: 10.1093/g3journal/jkaf109 (PMC12239632; doi:10.1093/g3journal/jkaf109)
Supplement: jkaf109_Supplementary_Data [file jkaf109_supplementary_data.pdf]

Supplemental Material

Supplemental Tables

Table S1. Twelve mitochondrial genomes downloaded from Genbank (Hassanin et al, 2012) used for the reconstruction of the mitochondrial genome phylogeny (Figure 5).

Scientific name	common name	Tissue Origin	NCBI accession number
Tragelaphus angasii	Nyala	MBP15†, South Africa	JN632702*
Tragelaphus buxtoni	Mountain nyala	-	NC_038064
Tragelaphus derbianus	Giant eland	SPOT 4178, RCA	EF536354*
Tragelaphus eurycerus	Bongo	SUN 8, Congo	JN632703*
Tragelaphus imberbis	Lesser kudu	PhC15, Tanzania	EF536356*
Tragelaphus oryx	Common eland	PhC13, Tanzania	JN632704*
Tragelaphus scriptus isolate1	Bushbuck	CAR31†, Cameroon	JN632705*
Tragelaphus scriptus isolate2	Bushbuck	MBP11†, South Africa	JN632706*
Tragelaphus scriptus isolate3	Bushbuck	PhC17, Tanzania	JN632707*
Tragelaphus spekii	Sitatunga	GLC6, Gabon	EF536357*
Tragelaphus strepsiceros	Greater kudu	PhC11, Tanzania	JN632708*
Syncerus caffer	African buffalo	PhC22, Tanzania	EF536353*
Tragelaphus eurycerus isaaci	Mountain bongo	*ex-situ* USA - EDTA blood	This studyta

† Samples collected from local hunters, suggesting the possibility of species misidentification (Hassanin et al 2012)

*Sequenced by Hassanin et al 2012 and downloaded from NCBI.

Table S2. Comparison of BUSCO completeness scores and percentage of repetitive elements masked using RepeatMasker for the genomes of five *Bovidae* species and the okapi (*Giraffidae*).

Species	Complete BUSCOs	% masked	Resource
**domestic cattle (*Bos taurus*)**	90.8%	46.50%	(Adelson et al., 2009)
**Sable (*Hippotragus niger*)**	94.8%	46.97%	(Koepfli et al., 2019)
**Gemsbok (*Oryx gazella*)**	96.3%	41.27%	(Farré et al., 2019)
**scimitar-horned oryx (*Oryx dammah)***	93.3%	47.80%	(Humble et al., 2020)
**Roan antelope (*Hippotragus equinus*)**	91.2%	42.20%	(Gonçalves et al., 2021)
**Okapi (*Okapia johnstoni*)**	94.3%	43.53%	(Winter et al., 2022)

References:

Adelson, D.L., Raison, J.M., Edgar, R.C., 2009. Characterization and distribution of retrotransposons and simple sequence repeats in the bovine genome. Proc Natl Acad Sci U S A 106, 12855–12860. https://doi.org/10.1073/pnas.0901282106

The additional references in this table are found in the main text reference list.

Supplemental Methods:

Computational scripts for the assembly of the 10X Genomics Chromium linked-reads using Supernova v2.1.1.

```
supernova run --id=barney_sn2 --fastqs=./ --description=supernova2_2019-5-23 --maxreads=1094007643 --
localcores=10


supernova mkoutput --asmdir=./barney_sn2/outs/assembly \
--outprefix=barney_pseudo \
--style=pseudohap
```
